# Radiation Safety and Hormesis

**DOI:** 10.3389/fpubh.2020.00278

**Published:** 2020-07-24

**Authors:** Sergei V. Jargin

**Affiliations:** Department of Public Health, Peoples' Friendship University of Russia, Moscow, Russia

**Keywords:** ionizing radiation, hormesis, radiation safety, aerospace health, East Urals Radioactive Trace

This is a summarizing update of several preceding publications (1, 2) with special reference to the series of papers concerning the East Urals Radioactive Trace (EURT) (3–10). A realistic risk assessment and potential hormetic effects are of particular importance for the aerospace health and safety, where exposures to ionizing radiation may be unavoidable but not necessarily harmful. Hormesis is a biphasic dose response: low doses exert protective effects while higher doses are detrimental. Among environmental factors acting according to the hormetic pattern are numerous physical and chemical agents including products of water radiolysis (11, 12). In the hormesis framework, mild exposures to various stressors elicit an adaptive response that enhances defenses and protects the organism. This protection may be associated with a performance increase that goes beyond that observed in untreated individuals (13). The term “hormesis” is rarely mentioned in publications on the field of radiation protection while the linear no-threshold (LNT) hypothesis is continued to be used. According to the LNT, the probability of developing cancer is proportional to the radiation dose; the dose-response relationships can be extrapolated down to low doses, where the correlations are unproven and can become inverse in accordance with the hormesis concept. In fact, to reject the LNT hypothesis, it would suffice to prove hormesis (14). The LNT is based on the following premise: the more particle tracks go through cell nuclei, the more DNA damage would occur and the higher the damage would be. This concept does not take into account that DNA damage and repair are permanent processes in the dynamic equilibrium. By analogy with other environmental factors, an evolutionary adaptation to the natural background radiation can be expected. The conservative nature of the DNA repair suggests that cells and organisms may have retained some capability of repair from a higher radiation impact than that caused by the present background. Life evolved on the Earth with a radiation background that was higher than that existing today (15, 16). The experimental evidence in favor of hormesis and adaptive responses to radiation is considerable (13, 17, 18); more details and references are in (1, 2, 19). This signifies that experimental data partly disagree with epidemiological studies. The main problem with the epidemiological research of low-dose radiation is potential bias, inter-study differences in quality, and reliability (1, 20). Of particular significance is selection and self-selection: persons with higher doses or those residing in more contaminated areas would be averagely more preoccupied with their health, compensations, and provisions, being at the same time given more attention, the diagnostic quality thus being potentially dose-dependent. In case-control studies, the cases tend to recollect circumstances related to exposures better than controls (recall bias), which may result in dose-effect correlations (1, 21).

Hormetic responses to therapeutics as compared to radiation hormesis should be briefly commented. Radiation is an environmental factor. There are no a priori grounds to expect biphasic dose-responses for factors that are absent in the natural environment. This general principle does not exclude a possibility that some substances, if even absent in the natural environment, can act according to the hormetic pattern due to some known or unknown mechanisms (13, 22–24). Microorganisms may develop hormetic responses to antibiotics by a positive selection of resistant strains. The theoretic basis of some hormetic mechanisms was discussed within the framework of stress response pathways (13, 22). Of note, different kinds of stress are a part of the environmental impact on living organisms, the latter being accordingly adapted. On the other hand, some pharmacological and toxicological stimuli can have a cumulative effect or act synergistically with some noxious factors, for example, upon cells with a limited or lacking mitotic capacity such as cardiomyocytes or neurons. It can be of particular importance in conditions when such cells are pre-damaged, e.g., by ischemia, so that even a minor additional impact may act according to the no-threshold dose-response pattern without hormesis. Under such circumstances, which are common especially in gerontology, the hormesis concept can be precarious if used in the clinical decision-making. All clinically significant effects, hormetic or not, should be tested according to the principles of evidence-based medicine (25).

Along with the elevated cancer risk, an increased risk of non-cancer outcomes has been reported, e.g., in the EURT cohorts (3–9). For example, the incidence of cerebrovascular diseases (CVD) was significantly elevated among Mayak facility workers with accumulated external gamma-ray doses ≥0.2 Gy compared to those exposed to lower doses (3). The risk estimate in (3) was higher than in other studies. Nonetheless, in a later publication, a significantly enhanced CVD risk was reported for the doses as low as ≥0.1 Gy (4). The excess relative risk of CVD per 1 Gy in Mayak workers turned out to be even higher than among atomic bomb survivors (5, 6), where the exposure was acute and hence presumably more efficient per dose unit. Elevated risks compared to those calculated using the LNT model have been found in the Techa River cohort for the total of cardiovascular diseases and separately for ischemic heart disease (7). The average total cumulated gamma-ray dose to male Mayak workers studied in (6, 8) was ~0.91 Gy while ≥90% of the Techa river cohort received ≤0.1 Gy (7). For comparison, an increased risk of heart disease in patients receiving radiation therapy has been associated with mediastinal doses ≥40 Gy and breast doses 40–50 Gy (26), i.e., fractionated but still high-dose-rate exposures. According to the BEIR VII (Biologic Effects of Ionizing Radiation) Report, radiation has been demonstrated to increase the risk of diseases other than cancer, particularly cardiovascular disease, in patients exposed to high therapeutic doses and in A-bomb survivors exposed to more modest doses. However, there is no direct evidence of increased risk of non-cancer diseases at low doses (27). According to the United Nations Scientific Committee on the Effects of Atomic Radiation (UNSCEAR), existing evidence is not sufficient to establish a causal relationship between ionizing radiation and cardiovascular disease at doses ≤1–2 Gy (26). The threshold may be underestimated due to bias in the epidemiological research. Doses associated with cardiovascular damage in experiments have been generally higher than averages in human populations exposed due to accidents and other anthropogenic contaminations, overviewed in (1, 2). Reported dose-effect relationships between low-dose low-rate exposures and non-neoplastic diseases cast doubt on such relationships for cancer found by the same researchers.

Certain reports on the enhanced cancer risk appear doubtful. For example, a significant increase in the skin cancer risk was found among Mayak et al. (10). A bias was not excluded: the workers and medical personnel knew individual work histories wherefrom cumulated doses could be estimated possibly influencing the diagnostic thoroughness and self-reporting. Skin doses were unknown (10). The workers were exposed predominantly to gamma rays having a relatively high penetration distance, so that the portion of energy absorbed by the skin must have been correspondingly low. Not surprisingly, the “pre-malignant skin lesions and actinic keratoses… were very rare in members of the study cohort” (10). Other questionable results and conclusions have been cited previously (2), for example: “CVD incidence was significantly higher among workers with total absorbed external γ-ray doses greater than 0.1 Gy compared to those exposed to lower doses and that CVD incidence was also significantly higher among workers with total absorbed internal alpha-particle doses to the liver from incorporated plutonium greater than 0.01 Gy compared to those exposed to lower doses” (9). Considering the dose comparisons above and in (1), such results are probably caused by bias. Finally, unverified LNT-based mathematical models have been applied to the EURT data contributing to the overestimation of medical consequences of low-dose low-rate exposures to ionizing radiation (28) commented previously (29). Potential motives have been discussed elsewhere (1, 2).

## Conclusion

The monitoring of populations exposed to low-dose low-rate radiation is important but will hardly add much reliable information on the health risks. It can be reasonably assumed that the screening, increased attention of exposed people to their health, and biased research will result in new reports on the elevated detection rate of cancer and other diseases in exposed populations. A constructive alternative for the future work would be large-scale animal experiments. The life duration is known to be a sensitive endpoint attributable to radiation exposures. Low-dose exposures were reported to extend the lifespan of mice and some invertebrates (13). Cardiovascular effects and hormesis could be measured in chronic experiments applying exercise tolerance (time to fatigue) and other functional tests. The spectrometry describing changes in the heart proteome may provide valuable information. Promising data in favor of radiation hormesis have been received also in the research of brain tissues (18). Moreover, the question should be clarified in cell cultures and *in vivo* whether relevant doses cause an increase in cell death, i.e., apoptosis. To enable extrapolations to humans, the doses and dose rates in experiments must be comparable to those in corresponding human populations, taking into account the radiosensitivity and life span of given species. Further experiments with different animal species would contribute to a better quantification of their radiosensitivity, thus enabling more precise extrapolations to humans.

## Author Contributions

The author confirms being the sole contributor of this work and has approved it for publication.

## Conflict of Interest

The author declares that the research was conducted in the absence of any commercial or financial relationships that could be construed as a potential conflict of interest.

## References

[1] Jargin SV The Overestimation of Medical Consequences of Low-Dose Exposure to Ionizing Radiation. Newcastle upon Tyne: Cambridge Scholars Publishing (2019).

[2] Jargin SV. Hormesis and radiation safety norms: comments for an update. Hum Exp Toxicol. (2018) 37:1233–43. 10.1177/0960327118765332 29607734

[3] Azizova TV Muirhead CR Moseeva MB Grigoryeva ES Sumina MV O'Hagan J. Cerebrovascular diseases in nuclear workers first employed at the Mayak PA in 1948-1972. Radiat Environ Biophys. (2011) 50:539–52. 10.1007/s00411-011-0377-6 21874558

[4] Azizova TV Haylock R Moseeva MB Pikulina MV Grigorieva ES. Cerebrovascular diseases incidence and mortality in an extended Mayak worker cohort: 1948-1982. Med Radiol Radiat Saf . (2015) 60:43–61. Available online at: http://medradiol.fmbafmbc.ru/journal_medradiol/abstracts/2015/4/43_Azizova.pdf 25361397 10.1667/RR13680.1

[5] Azizova TV Muirhead CR Druzhinina MB Grigoryeva ES Vlasenko EV Sumina MV Cerebrovascular diseases in the cohort of workers first employed at Mayak PA in 1948-1958. Radiat Res. (2010) 174:851–64. 10.1667/RR1928.1 21128809

[6] Moseeva MB Azizova TV Muirhed CR Grigor'eva ES Vlasenko EV Sumina MV. Risk of cerebrovascular disease incidence in the cohort of Mayak production association workers first employed during 1948-1958. Radiats Biol Radioecol. (2012) 52:149–57. 22690577

[7] Krestinina LY Epifanova S Silkin S Mikryukova L Degteva M Shagina N. Chronic low-dose exposure in the Techa River cohort: risk of mortality from circulatory diseases. Radiat Environ Biophys. (2013) 52:47–57. 10.1007/s00411-012-0438-5 23124827

[8] Azizova TV Muirhead CR Druzhinina MB Grigoryeva ES Vlasenko EV Sumina MV Cardiovascular diseases in the cohort of workers first employed at Mayak PA in 1948-1958. Radiat Res. (2010) 174:155–68. 10.1667/RR1789.1 20681782

[9] Azizova TV Haylock RG Moseeva MB Bannikova MV Grigoryeva ES. Cerebrovascular diseases incidence and mortality in an extended Mayak Worker Cohort 1948-1982. Radiat Res. (2014) 182:529–44. 10.1667/RR13680.1 25361397

[10] Azizova TV Bannikova MV Grigoryeva ES Rybkina VL Risk of malignant skin neoplasms in a cohort of workers occupationally exposed to ionizing radiation at low dose rates. PLoS ONE. (2018) 13:e0205060 10.1371/journal.pone.0205060 30289933 PMC6173419

[11] Sanders CL Radiobiology and Radiation Hormesis: New Evidence and its Implications for Medicine and Society. Cham: Springer Nature; Springer International Publishing AG (2017).

[12] Kaludercic N Deshwal S Di Lisa F. Reactive oxygen species and redox compartmentalization. Front Physiol. (2014) 5:285. 10.3389/fphys.2014.00285 25161621 PMC4130307

[13] Berry R III López-Martínez G. A dose of experimental hormesis: when mild stress protects and improves animal performance. Comp Biochem Physiol A Mol Integr Physiol. (2020) 242:110658. 10.1016/j.cbpa.2020.110658 31954863 PMC7066548

[14] Cardarelli JJ II Ulsh BA. It is time to move beyond the linear no-threshold theory for low-dose radiation protection. Dose Response. (2018) 16:1559325818779651. 10.1177/1559325818779651 30013457 PMC6043938

[15] Karam PA Leslie SA. Calculations of background beta-gamma radiation dose through geologic time. Health Phys. (1999) 77:662–7. 10.1097/00004032-199912000-00010 10568545

[16] Sugimoto M Hormesis: Insight into adaptive defense mechanisms against ionizing radiation established during evolution of life on the Earth. In: Sutou S, editor. Fukushima Nuclear Accident. New York, NY: Nova Publishers (2015). p. 89–100.

[17] Tapio S Jacob V. Radioadaptive response revisited. Radiat Environ Biophys. (2007) 46:1–12. 10.1007/s00411-006-0078-8 17131131

[18] Hladik D Dalke C von Toerne C Hauck SM Azimzadeh O Philipp J. CREB signaling mediates dose-dependent radiation response in the murine hippocampus two years after total body exposure. J Proteome Res. (2020) 19:337–45. 10.1021/acs.jproteome.9b00552 31657930

[19] Luckey TD. Radiation hormesis: the good, the bad, and the ugly. Dose Response. (2006) 4:169–90. 10.2203/dose-response.06-102 18648595 PMC2477686

[20] Little MP Tawn EJ Tzoulaki I Wakeford R Hildebrandt G Paris F. Review and metaanalysis of epidemiological associations between low/moderate doses of ionising radiation and circulatory disease risks, and their possible mechanisms. Radiat Environ Biophys. (2010) 49:139–53. 10.1007/s00411-009-0250-z 19862545 PMC3075616

[21] Jorgensen TJ. Dental x-rays and risk of meningioma. Cancer. (2013) 119:463. 10.1002/cncr.27710 23254822

[22] Calabrese V Cornelius C Dinkova-Kostova AT Calabrese EJ Mattson MP. Cellular stress responses, the hormesis paradigm, and vitagenes: novel targets for therapeutic intervention in neurodegenerative disorders. Antioxid Redox Signal. (2010) 13:1763–811. 10.1089/ars.2009.3074 20446769 PMC2966482

[23] Calabrese V Giordano J Crupi R Di Paola R Ruggieri M Bianchini R. Hormesis, cellular stress response and neuroinflammation in schizophrenia: early onset versus late onset state. J Neurosci Res. (2017) 95:1182–93. 10.1002/jnr.23967 27898171

[24] Calabrese EJ Bhatia TN Calabrese V Dhawan G Giordano J Hanekamp YN. Cytotoxicity models of Huntington's disease and relevance of hormetic mechanisms: a critical assessment of experimental approaches and strategies. Pharmacol Res. (2019) 150:104371. 10.1016/j.phrs.2019.104371 31415915

[25] Jargin SV. Hormesis: umbrella mechanism only for agents present in the environment. Hum Exp Toxicol. (2015) 34:439–41. 10.1177/0960327114564796 25810501

[26] UNSCEAR 2006 Report to the General Assembly Annex B: Epidemiological Evaluation of Cardiovascular Disease and Other Noncancer Diseases Following Radiation Exposure. New York, NY: United Nations (2006).

[27] National Research Council of the National Academies Health Risks from Exposure to Low Levels of Ionizing Radiation: BEIR VII Phase 2. Washington, DC: The National Academies Press (2006) p. 245 10.17226/11340

[28] Smirnova OA Akleyev AV Dimov GP. Analysis of hematopoiesis dynamics in residents of Techa riverside villages chronically exposed to nonuniform radiation: modeling approach. Health Phys. (2014) 106:445–58. 10.1097/HP.0b013e3182a81d2c 24562065

[29] Jargin SV Focused review of mathematical modeling of radiation-related abnormalities in the Techa River cohort. J Environ Occup Health. (2015) 4:114–7. 10.5455/jeos.20150312010516

